# Exosomes for Intramyocardial Intercellular Communication

**DOI:** 10.1155/2015/482171

**Published:** 2015-05-21

**Authors:** Elisabetta Cervio, Lucio Barile, Tiziano Moccetti, Giuseppe Vassalli

**Affiliations:** ^1^Molecular Cardiology Laboratory, Fondazione Cardiocentro Ticino, Via Tesserete, 6900 Lugano, Switzerland; ^2^Swiss Institute of Regenerative Medicine (SIRM), Taverne, Switzerland; ^3^Centre Hospitalier Universitaire Vaudois (CHUV), Avenue du Bugnon, 1011 Lausanne, Switzerland

## Abstract

Cross-talk between different cell types plays central roles both in cardiac homeostasis and in adaptive responses of the heart to stress. Cardiomyocytes (CMs) send biological messages to the other cell types present in the heart including endothelial cells (ECs) and fibroblasts. In turn, CMs receive messages from these cells. Recent evidence has now established that exosomes, nanosized secreted extracellular vesicles, are crucial mediators of such messages. CMs, ECs, cardiac fibroblasts, and cardiac progenitor cells (CPCs) release exosomes carrying nonrandom subsets of proteins, lipids, and nucleic acids present in their cells of origin. Exosomes secreted from CMs are internalized by fibroblasts and regulate gene expression in these cells as well as in ECs. CPC-derived exosomes protect CMs against apoptosis while also stimulating angiogenesis. They are rich in cardioprotective and proangiogenic microRNAs such as miR-146, miR-210, and miR-132. When injected into infracted hearts *in vivo*, CPC-derived exosomes reduce infarct size and improve cardiac function. Thus, exosomes are emerging both as key mediators of intercellular communication in the heart and as therapeutic candidates for heart disease.

## 1. Introduction

While cardiomyocytes (CMs) make up a large part of the volume of the heart, fibroblasts are the most abundant cell type that accounts for ~90% of the nonmyocyte cells present within the heart [1]. Endothelial cells (ECs) and vascular smooth muscle cells (VSMCs) are the other major cell types present in the heart. Moreover, accumulating evidences suggest that the adult mammalian heart may harbor a pool of resident stem and cardiac progenitor cells (CPCs) that participate in cardiac responses to injury and possibly in physiological CMs turnover during aging [2, 3]. Cellular cross talk plays a central role both in cardiac homeostasis and in adaptive responses of the heart to stress. Chronic adaptive responses to stress, referred to as cardiac remodeling, include interstitial fibrosis, CMs hypertrophy, and changes in contractility and blood vessel density. Cells interact through direct contact as well as through secreted molecules, such as growth factors and cytokines, which bind to surface receptors of target cells and activate intracellular molecular signaling pathways downstream of these receptors. Aside from these well-established modalities of intercellular communication, emerging mechanisms include secreted extracellular vesicles (EVs) and circulating RNA. Secreted EVs carry payloads of proteins, lipids, and nucleic acids through interstitial fluid and circulating blood. They can be taken up by a variety of neighboring cells, as well as by cells at a distance [4, 5]. Growing evidence suggests that EVs may not only play a central role in the exchange of biological information between cells but may also be clinically useful both as biomarkers of cardiovascular diseases and as therapeutic candidates for these diseases [6, 7]. This review summarizes recent advances in our knowledge of the biological role of EVs, with a focus on exosomes (see below), in intercellular communication within the heart. The therapeutic potential of EV-based approaches in patients with heart disease will be briefly discussed.

## 2. Classes of EVs and Their Functional Roles

**Figure 1:**
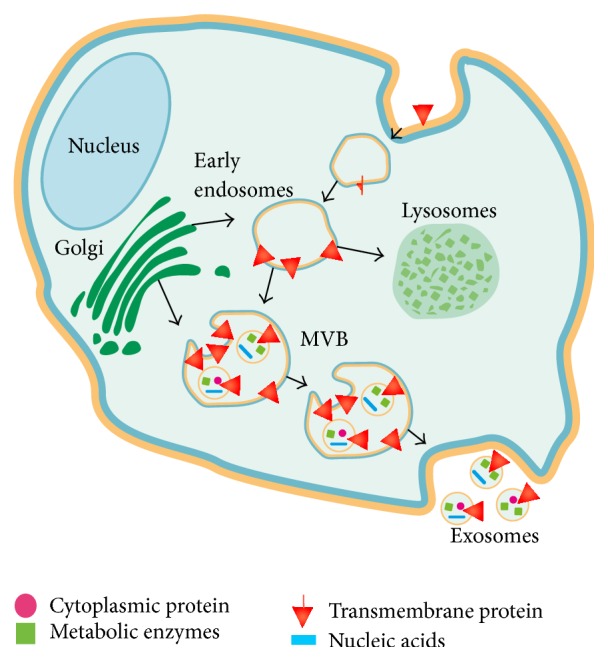
Biogenesis of exosomes. Exosomes are formed in the late endosomal compartment. They contain proteins from coated pits/lipid rafts in the cellular membrane, proteins directly sorted to the MVBs from RER and GC, mRNA, microRNA, and DNA. Exosomes are generated by inward budding of the limiting membrane of MVB, thus preserving the same orientation and folding of membrane-bound proteins on the exosomal membrane as those on the plasma membrane. The MVBs containing the exosomes either fuse with the plasma membrane to release exosomes or are sent to lysosomes for degradation (GC, Golgi complex; MVB, multivesicular body; and RER, rough endoplasmic reticulum).

EVs can be categorized based on their mechanisms of biogenesis and secretion. The major classes of EVs include exosomes, microvesicles, and apoptotic bodies. Exosomes are 40 to 150 nm diameter lipid vesicles that originate from the endolysosomal pathway and are assembled into multivesicular bodies (MVBs) that either traffic to the lysosome or fuse with the plasma membrane and release their contents of exosomes into the extracellular space [8, 9] (Figure 1). The mechanism that directs exosomes to lysosome versus extracellular space is poorly understood. Exosomes released from the producing cell can bind to the plasma membrane of the acceptor cell and be internalized through either endocytosis or micropinocytosis [10]. In contrast to exosomes, microvesicles typically range from 200 to 1000 nm in diameter and arise by direct budding from the plasma membrane [11]. Finally, apoptotic bodies range from 50 to 2000 nm in diameter and are generated through blebbing of the surface of apoptotic cells [12].

Originally described as the mechanism by which reticulocytes discard superfluous receptors and protein complexes as they mature to erythrocytes [13], exosomes are now recognized as important mediators of intercellular communication in many organs including the heart [14–16]. In cancer, for instance, exosomes can pass on malignancy from cell to cell and shape the environment by stimulating blood vessel formation while concomitantly inhibiting immune responses to the tumor [17–19]. Similar roles for exosomes have been proposed in viral infections [20, 21], as exosomes can shuttle viral particles from cell to cell.

Exosomes carry proteins, lipids, and nucleic acids including mRNA, DNA, and noncoding RNAs such as microRNAs (miRNAs) and long noncoding RNAs (lnRNAs) [22–24]. By targeting multiple transcripts in a coordinated manner, noncoding RNAs regulate many cell processes. As an example, miR-590 and miR-199a have been shown to reprogram adult CMs to proliferate [25]. Of note, it has been demonstrated that mRNAs contained in exosomes can be translated in acceptor cells and that miRNAs traveling with exosomes can inhibit gene expression and regulate biological functions in these cells [26, 27]. These findings indicate that exosomes can function as vehicles for the exchange of genetic information between cells [28].

It should be emphasized that the protein and RNA contents of exosomes are nonrandom subsets of the protein and RNA contents of their cells of origin [29]. The mechanisms that regulate the biogenesis of exosomes and incorporation of proteins and RNA into them are incompletely understood. The endosomal sorting complex required for transport (ESCRT) plays an important role in this regard. As an example, RNA interference screens targeting 23 components of the ESCRT machinery and associated proteins in MHC class II- (MHC II-) expressing HeLa-CIITA cells showed that silencing of HRS, STAM1, or TSG101 reduced the secretion of EV-associated CD63 and MHC II but each gene altered differently the size and/or protein composition of secreted EVs [30]. By contrast, depletion of VPS4B augmented this secretion but did not alter the features of EVs. Silencing of ALIX increased MHC II exosomal secretion, as a result of an overall increase in intracellular MHC II protein and mRNA levels. These results revealed a role for selected ESCRT components and accessory proteins in exosome secretion and composition.

The online compendium ExoCarta [31] provides an updated list of the biomolecules that have been found in exosomes. Exosome secretion by various cell types present in the heart including CMs, ECs, fibroblasts, and CPCs has been demonstrated. However, the role of these EVs in cellular cross talk and cardiac homeostasis remains to be fully elucidated.

## 3. Exosomes Released from CMs Regulate Cardiac Fibroblast and EC Functions

In 2007, Gupta and Knowlton [32] reported, for the first time, that adult CMs release exosomes. Using differential centrifugation and ultracentrifugation techniques, they were able to isolate EVs, which were then categorized as exosomes, from primary cultures of adult rat CMs. EVs were secreted by CMs under baseline conditions, but brief hypoxia increased their release. They contained heat shock protein 60 (Hsp60) which, unlike Hsp70 and Hsp90, had not been previously described in EVs from other cell types. It has been suggested that extracellular Hsp60, when not in exosomes, may trigger apoptosis in CMs via activation of Toll-like receptor (TLR)4 [33]. The population of EVs secreted by CMs was not homogeneous, ranging in diameter from 40 to 300 nm. The exosomal marker flotillin-1 was detectable in 80% of EVs. Proteomics analyses revealed that the protein content of exosomes released from primary adult CMs included cytosolic, sarcomeric, and mitochondrial proteins, differing significantly from other types of exosomes described in the literature. These proteins included myomesin, myosin-binding protein C, VCP (also known as p97 AAA-ATPase, Cdc48, and transitional endoplasmic reticulum ATPase), tropomyosin, and *α*-crystallin [33]. Ethanol, at concentrations seen with the consumption of alcoholic beverages, increased the release of exosomes from CMs. Ethanol-treated cells showed enhanced Cell-Rox red staining, consistent with a mechanistic role of reactive oxygen species (ROS) in the release of exosomes from CMs. Hypoxia/reoxygenation also modified the protein content of exosomes released by CMs.

The interplay of CMs and cardiac fibroblasts plays important roles in adaptive responses of the heart to increased workload as a result of myocardial infarction (MI), hypertension, or heart valve disease [34]. These responses include hypertrophy, changes in contractility, and interstitial fibrosis. This cellular interplay may result in CMs growth or possibly proliferation, as well as changes in fibroblast function that affect extracellular matrix. Waldenström et al. [35] isolated EVs from media of cultured HL-1 cells, an immortalized atrial myxoma cell line, by differential centrifugation including preparative ultracentrifugation. Isolated EVs were surrounded by a bilayered membrane and flow cytometry revealed presence of both caveolin-3 and flotillin-1. Out of 1520 detected 2 mRNA, 423 could be directly connected in a biological network. Moreover, exosomal DNA transfer into fibroblasts was demonstrated. Exosomes stained for DNA were seen in the fibroblasts cytosol and even in the nuclei. Changes in gene expression were detected in NIH-3T3 fibroblasts transfected by CM-derived EVs. Among 333 gene expression changes, there were 175 upregulations and 158 downregulations compared with controls. These findings demonstrate that exosomes can transfer genetic information from CMs to fibroblasts and affect their transcript profiles. Clearly, these data in NIH-3T3 fibroblasts cultured in the presence of exosomes released by HL-1 cells cannot be directly translated to adult CM-derived exosomes and cardiac fibroblasts.

Another study by the same group analyzed mRNA profiles of exosomes released from HL-1 cells, under baseline and stimulated conditions [36]. The presence of transforming growth factor- (TGF-) *β*2 or platelet-derived growth factor- (PDGF-) BB in cell culture media altered the transcript profiles of the exosomes. Common transcripts (217) were found in all 3 groups. The number of transcripts detected in the TGF-*β*2-treated group and the PDGF-BB-treated group was 562 and 300, respectively, as compared to 505 in the control group.

Yu et al. [37] reported that prolonged hypoxia increased levels of tumor necrosis factor- (TNF-) *α* in exosomes secreted by cultured neonatal CMs. They provided evidence suggesting that, under hypoxia, hypoxia inducible factor- (HIF-) 1*α* initiates expression of TNF-*α*, mediated by exosomes in CMs. Zhang et al. [38] showed that Hsp20 (HspB6), a small heat shock protein that is increased in blood from cardiomyopathic hamsters, was secreted by cultured CMs through exosomes and promoted angiogenesis. These findings show that external stimuli can modify the molecular content of exosomes secreted by CMs.

Recent evidence suggests CMs may interplay with cardiac ECs through exosomes. Wang et al. [39] reported that exosomes secreted by CMs from adult Goto-Kakizaki rats, a commonly used animal model of type 2 diabetes, inhibited proliferation, migration, and tube-like formation in cocultured mouse cardiac ECs, whereas exosomes secreted by CMs from control Wistar rats induced the opposite effects. Exosomes from CMs from Goto-Kakizaki rats contained higher levels of miR-320 but lower levels of miR-126, compared to those from control rats, and these differences explained most of the observed effects on cardiac ECs. Indeed, miR-320 transfer into cardiac ECs using CM exosomes from Goto-Kakizaki rats downregulated target genes of miR-320, such as insulin-like growth factor- (IGF-) 1, Hsp20, and Ets2. Reciprocally, knocking down of miR-320 abolished the antiangiogenic effects of the exosomes. These data suggest exosome-mediated miR-320 transfer from CMs into cardiac ECs may inhibit angiogenesis. This mechanism could participate in the development of diabetic coronary microvascular disease.

## 4. Exosomes Released from ECs Regulate VSMC, CPC, EC, and B Cell Functions

In the heart, ECs and CMs interact to match oxygen and nutrient demand and supply. Acting in concert with endothelial progenitor cells (EPCs) and stromal cells, ECs participate in the maintenance of vascular integrity. It has been shown that ECs release EVs including exosomes and that TNF-*α* enhances intercellular adhesion protein-1 (ICAM-1) and mRNA expression in these exosomes [40]. This observation suggests that cellular stress may affect the protein and RNA profiles of exosomes secreted by ECs, which implies a role for exosomes in the propagation of stress between vascular cells, and that analysis of the protein and RNA profiles of EC-derived exosomes may provide information regarding vascular stress. Another study by van Balkom et al. [41] showed that EC-derived exosomes stimulate migration and angiogenesis in target ECs in a miR-214-dependent manner. Moreover, Hergenreider et al. [42] described an EV-mediated, atheroprotective communication between ECs and VSMCs involving miRNA transfer.

Ong et al. [43] recently showed that exosomes released by cardiac ECs are actively internalized by CPCs* in vitro*. ECs transfected with the HIF-1 gene secreted exosomes enriched for miR-210 and miR-126. CPCs cultured in the presence of exosomes from HIF-1-transfected ECs activated prosurvival kinases and exhibited a glycolytic switch. In a NOD/SCID mouse model of MI, CPC transplantation and intramyocardial injection of nonviral microcircle plasmid carrying the HIF-1 gene induced synergistic beneficial effects that were mediated by miR-210 and miR-126. These findings are consistent with the notion that EC-derived exosomes can exert functional effects on CPCs.

Recently, Halkein et al. [44] reported that 16-kDa N-terminal prolactin fragment, cleaved from the full-length nursing hormone prolactin by cathepsin D, not only induced the expression of miR-146a in ECs, leading to inhibition of angiogenesis, but also enhanced the release of miR-146a-enriched exosomes from ECs. These EC-derived exosomes could be taken up by CMs, resulting in increased miR-146a levels and reduced expression of Erbb4, Notch1, and Irak1 (targets of miR-146a) in CMs, resulting in impaired metabolic activity and contractile function. These findings confirm a miRNA-based intercellular communication system between ECs and CMs through exosomes, while suggesting a role for exosomes in peripartum cardiomyopathy.

Finally, Song et al. [45] reported that exosomes released from murine cardiac ECs carried integrin *α*v*β*6 that converted the latent TGF-*β* expressed by B cells upon lipopolysaccharide (LPS) stimulation to the active form, TGF-*β*. Thus, B cells released TGF-*β* in response to reexposure to cardiac EC-derived exosomes in the culture, inducing less proliferation of effector T cells. These findings suggest that EC-derived exosomes may favor the generation of B cells with immune suppressor functions.

## 5. Exosomes Released from Cardiac Fibroblasts Regulate CM Functions

Cardiac fibroblasts regulate myocardial function via soluble mediators in a paracrine manner [46, 47]. A key regulator of cellular interactions between fibroblasts and CMs is TGF-*β* [48]. Bang et al. [49] recently showed that exosomes derived from cardiac fibroblasts contained relatively high levels of many miRNA passenger strands (“star” miRNAs) that normally undergo intracellular degradation, such as miR-21-3p (miR-21^*^). They also showed that miR-21^*^ is a potent stimulator of CM hypertrophy through regulation of its target genes sorbin and SH3 domain-containing protein 2 (SORBS2) as well as PDZ and LIM domain 5 (PDLIM5). Silencing SORBS2 or PDLIM5 in CMs induced hypertrophy, whereas pharmacological blockade of miR-21^*^ attenuated pathology in a mouse model of angiotensin-II-induced cardiac hypertrophy. These findings suggest a role for exosomes released from cardiac fibroblasts in the development of CMs hypertrophy.

## 6. Exosomes Released from Mesenchymal Stem Cells (MSCs) and Bone Marrow CD34^+^ Cells Regulate CM and EC Functions

Timmers et al. [50] reported that MSC conditioned medium administered intravenously just before myocardial reperfusion after ischemia reduced infarct size in pigs and mice. Exosomes from embryonic MSCs injected systemically before reperfusion after coronary ligation similarly reduced infarct size in mice [51]. Using a gene therapy approach, exosomes secreted from GATA-4 overexpressing MSCs served as a reservoir of antiapoptotic miRNAs for cardioprotection [52].

Bone marrow CD34^+^ stem cells exhibit angiogenic paracrine activities. Sahoo et al. [53] identified exosomes as the active component of these activities. CD34^+^ stem cell-derived exosomes injected into ischemic mouse hearts were selectively internalized by ECs and CMs but not by fibroblasts. These exosomes improved cardiac function and capillary density while reducing fibrosis* in vivo*. To sum up, growing evidence suggests that exosomes derived from various stem cell types such as MSCs and bone marrow CD34^+^ cells may possess cardioprotective and proangiogenic activities.

## 7. Exosomes Released from CPCs Are Cardioprotective and Proangiogenic and Improve Cardiac Function

**Figure 2:**
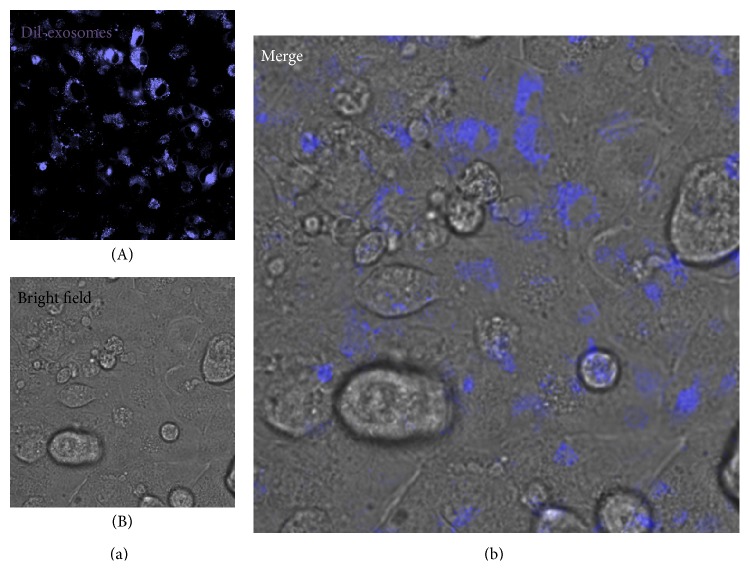
CPC exosome uptake by HL-1 CMs. (A) Laser confocal fluorescence photomicrograph showing HL-1 cells cultured with Dil-labeled (blue) human CPC exosomes. (B) Corresponding bright photomicrograph. (b) Merge (high magnification).

Recently, Sahoo and Losordo [16] provided ultrastructural evidence of exosome-like vesicles packed in MVBs of a CPC, characterized by a large nucleus and a thin cytoplasm, in a healthy mouse heart, as well as in the cytoplasm of CMs from the left ventricle of a healthy human and from a patient with ischemic heart disease. Using electron microscopy, we likewise provided ultrastructural evidence of exosome-like vesicles released by human cardiospheres* in vitro* (i.e., cellular aggregates formed by CPCs cultured in cardiosphere-forming medium under low-adhesion conditions [54, 55]) and by cells in the mouse heart* in vivo* [56]. We also demonstrated internalization of human CPC-derived exosomes by HL-1 CMs* in vitro* (Figure 2). These exosomes were enriched for several cardioprotective and proangiogenic miRNAs, such as miR-210, miR-132, and miR-146 compared with exosomes secreted by normal human dermal fibroblasts (NHDFs) [57]. HL-1 cells cultured in the presence of CPC-derived exosomes showed a time-dependent increase in the intracellular concentrations of miR-210 and miR-132 suggesting uptake of CPC exosomes by these cells. On the other hand, Gray et al. [58] reported that mouse CPC-derived exosomes were internalized efficiently by cardiac fibroblasts but poorly by CMs. These differences could be explained by species-specific differences in the CPCs used as exosome donors as well as in target CMs. Exosomes secreted by mouse CPCs in response to hypoxia enhanced ECs tube formation while reducing profibrotic gene expression in TGF-*β*-stimulated fibroblasts. Vrijsen et al. [59] showed that CPC-derived exosomes stimulated the migration of ECs, in part due to their matrix metalloproteinase (MMP) content.

**Figure 3:**
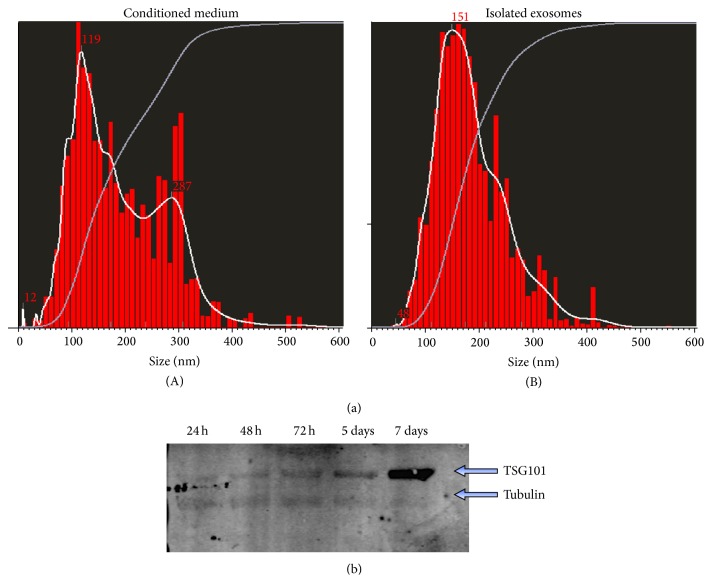
(a) Nanoparticle tracking analysis of CPC conditioned medium (A) and isolated CPC exosomes (B) using the Nanosight system. A minor peak at 287 nm seen in conditioned medium is not seen in purified exosomes. (b) Western blot analysis of the exosome marker TSG101 in exosomes isolated at the indicated time points of CPC cultures.

Chen et al. [60] reported that mouse CPC-derived exosomes attenuated CMs apoptosis in ischemic-reperfused mouse hearts. Recent reports by Ibrahim et al. [61] and by our own group [57] showed that exosomes from adult human CPCs protected ischemic myocardium and regenerated the heart after myocardial injury. Ibrahim et al. [61] employed cardiosphere-derived cells (CDCs). CPCs obtained from endomyocardial biopsies from the right ventricular aspect of the interventricular septum from healthy hearts of deceased tissue donors formed cardiospheres [54, 55], from which CDCs were derived. By contrast, we obtained CPCs from right atrial appendage specimens from patients who underwent heart valve surgery [57]. Therefore, CDCs and CPCs used in the two studies were related yet distinct populations [57, 61]. EVs isolated from CPCs expressed the exosome marker TSG101. Nanoparticle tracking analysis revealed that they were not a pure exosomal population but also contained a minor component of larger microvesicles (130–300 nm) (Figure 3). CPC-derived exosomes inhibited apoptosis in HL-1 cells induced by serum deprivation. Moreover they stimulated tube formation of human umbilical vein ECs (HUVECs). When injected into rat hearts at the time of MI, CPC-derived exosomes reduced CMs apoptosis and scar size, increased viable mass and the number of newly formed blood vessels, and improved cardiac function* in vivo*. Exosomes from NHDFs, a therapeutically inert cell type in this model, lacked these benefits. Human CDC-derived exosomes similarly reduced CMs apoptosis under stress conditions while promoting CMs proliferation and angiogenesis* in vitro* [61]. When injected into the infarct border zone of immunodeficient mice at the time of MI, CDC exosomes improved global heart function, reduced scar size, and increased viable mass and infarcted wall thickness compared with NHDF exosomes. In a different model, CDC exosomes injected at 21 days postinfarct, a time point at which myocardial scar is well established [62], still reduced CMs apoptosis and scar size while improving viable mass, microvessels density, and cardiac function. These findings using a model of late exosome administration after injury are consistent with genuine cardiac regeneration.

## 8. miRNA Are Key Mediators of Cardioprotective and Proangiogenic Effects of CPC or CDC Exosomes

Exosomes secreted by different cell types differ in their miRNA contents. By differential miRNA profiling, both us and Ibrahim et al. [61] identified miR-146a as one of the most highly enriched miRNA in CPC and CDC exosomes compared to NHDF exosomes [57, 61]. Moreover, miR-146a tissue levels were increased in hearts injected with CDC exosomes [61]. miR-146a exerted cardioprotective effects* in vitro*. miR-146a knockout (KO) mice exhibited impaired heart function, increased scar mass, decreased infarct wall thickness, and worse postinfarct remodeling compared to wild-type mice of the same strain. Injection of a miR-146a mimic at the time of infarction “rescued” miR-146a KO mice with respect to these changes. Moreover, miR-146a-deficient exosomes, generated by transfecting CDCs with a miR-146a hairpin inhibitor followed by exosome isolation, showed impaired ability to protect CMs against oxidant stress [61]. Human CPC- or CDC-derived exosomes [57, 61], as well as hypoxia-preconditioned mouse CPC exosomes [58], were enriched for miR-210, a miRNA that has been implicated in CM survival and stimulation of angiogenesis [57, 63]. CPC exosomes also were enriched for miR-132, which similarly participates in angiogenesis and vascular remodeling [57, 64]. Together, these results point to a key role for miRNA transfer in the cardioprotective activities of CPC- or CDC-derived exosomes. Of note, it has been demonstrated that miRNAs can mediate long-lasting benefits and fundamental changes in the microenvironment of injured tissue [65].

## 9. Exosomes Secreted by Immune Cells

Immune cells including dendritic cells, macrophages, and T cells secrete immunologically active exosomes that influence both physiological and pathological processes. The immunological activities of exosomes affect both innate and adaptive immunity and include antigen presentation, T cell activation, T cell polarization to regulatory T cells, immune suppression, and anti-inflammatory effects [66, 67]. While the majority of peripheral blood EVs are derived from platelets, mononuclear phagocytes, including macrophages, are the second most abundant population [68]. RNA molecules contained in the macrophage-derived EVs are transported to target cells, including ECs, fibroblasts, and monocytes, and they induce the differentiation of naive monocytes into macrophages. miR-223 transported by macrophage-derived EVs to target cells is functionally active [68] and promotes ECs apoptosis by advanced glycation end products by targeting the IGF-1 receptor [69]. Body fluid exosomes can promote secretion of inflammatory cytokines in monocytic cells via Toll-like receptor signaling [70]. miRNA-containing EVs play important roles in vascular inflammation and atherosclerosis, which are discussed elsewhere [71].

In the setting of acute MI, the balance of macrophage subtypes (M1 versus M2 polarized macrophages) migrating into the infarcted region affects myocardial healing [72–74]. In another context, exosomes from cancer cells were shown to modulate macrophage infiltration and M1/M2 polarization [75]. Therefore, exosomes released by macrophages migrating into the injured heart could affect infarct healing by promoting monocyte differentiation into macrophages and by influencing macrophage polarization.

## 10. Exosomes and Remote Ischemic Preconditioning

Remote ischemic preconditioning induced by cycles of transient limb ischemia and reperfusion is a powerful cardioprotective strategy. Its underlying mechanisms are not precisely known, however. Recently, it has been shown that remote ischemic conditioning attenuates LV remodeling and interstitial fibrosis in the boundary region of MI via exosome-mediated intercellular communication [76]. This effect was associated with increased expression of miR-29a, a key regulator of tissue fibrosis, in exosomes and the marginal area of MI. IGF-1 receptor, which signals in the cardioprotective IGF-1 signaling pathway, was highly expressed both in the exosomes and in remote noninfarcted myocardium after remote ischemic conditioning. Another study [77] showed that remote ischemic conditioning, unlike ischemia/reperfusion, increased miR-144 levels in mouse myocardium and miR-144 precursor in exosome pellets. Systemic treatment with miR-144 increased P-Akt, P-GSK3*β*, and P-p44/42 MAPK, inducing early and delayed cardioprotection with improved functional recovery and decreased infarct size similar to that achieved by remote ischemic preconditioning. Conversely, systemic administration of a specific antisense oligonucleotide reduced myocardial levels of miR-144 and abrogated cardioprotection by remote ischemic preconditioning. These results indicate a cardioprotective role of miR-144 transported by exosomes.

## 11. Exosomes as Biomarkers of Heart Disease

As pointed out above, EVs including exosomes may be clinically useful as biomarkers of cardiovascular diseases [6, 7, 78–80]. A comprehensive discussion of this issue is beyond the scope of this review. As an example, Matsumoto et al. [81] reported a significant association of serum levels of miR-192, particularly in exosomes, in patients who experienced development of heart failure within 1 year after MI, as compared to matched controls without subsequent cardiovascular events after discharge. miR-192 is a p53-responsive miRNA. Interestingly, the serum levels of two other p53-responsive miRNAs, miR-194 and miR-34a, also were coordinately increased with miR-192, suggesting that these miRNAs may function as circulating regulators of heart failure development via the p53 pathway. miR-133 and miR-328 are increased in plasma in patients after MI and therefore represent novel biomarkers of acute MI [82]. In this regard, it should be emphasized that a majority of plasma and blood miRNAs travel with EVs.

## 12. Potential Exosome-Based Therapeutic Approaches for Heart Disease

Exosomes have been evaluated in a few clinical trials of therapeutic vaccination for cancer [83, 84]. Exosomes used in these trials were isolated from the conditioned media of dendritic cells that had been loaded with tumor antigen* ex vivo*. They were administered in an autologous fashion to induce antitumor immunity. In some patients, they elicited minor inflammatory responses at the site of injection and low-grade fever. However, repeated administration of exosomes was safe. Collectively, initial clinical trials have demonstrated that exosome therapy is feasible and safe in humans.

Proof-of-principle studies in animal models have demonstrated benefits of CPC- or CDC-derived exosomes injected intramyocardially [57, 58, 61], as mentioned above. Although CPCs remain a good cell candidate, the most effective cell type as a producer of cardioprotective exosomes remains to be identified. Cell manipulations aiming at enhancing beneficial properties of exosomes may include hypoxic and stress preconditioning [58], genetic modification [84], and epigenetic reprogramming of exosome-producing cells [85]. As cell-free therapeutic candidates, exosomes offer several potential advantages over cell therapy. An important question is whether allogeneic exosomes can be safely used in human. Exosomes carry MHC I and/or II, depending on the expression pattern of these molecules in their cells of origin. While allogeneic CDCs can be used in any recipient without adverse safety effects in animal models, some recipients develop low-level immune responses that may undermine the efficacy of subsequent administrations of identical biomaterial. In a recent study aiming at comparing the immunogenicity of repeated doses of xenogeneic (human) and allogeneic CDCs and CDC-derived EVs, subcutaneous injections of CDCs and CDC-derived EVs were delivered in rats every 2 weeks for up to 3 repeats [86]. As expected, repeated human (xenogeneic) CDC injections resulted in rapid humoral and cell-mediated immune responses, whereas delivery of human CDC-derived EVs did not induce any response after the first dose. Repeated dosing led to progressively increased immunogenicity; however, the response was diminished compared to that observed with human CDCs. Importantly, delivery of rat (allogeneic) CDC-derived EVs did not elicit any significant immune response even after repeated dosing. These results indicate that allogeneic CDC-derived EV delivery without immunosuppression elicits no overt immunogenicity after repeated dosing.

## 13. Conclusions

We have concisely discussed current evidence that exosomes play central roles in exchanges of biological information between cells, with a focus on the cross talk between the major cell types present in the heart. We have summarized recent data on cardioprotective and proangiogenic activities of exosomes secreted by human adult CPCs and CDCs. These exosomes are rich in miRNAs endowed with cardioprotective and regenerative potential. Although we often think of stem cell therapy acting to regenerate tissue through cell replication and then differentiation, growing evidence suggests that adult stem cells may exert dramatic effects in the repair of various tissues through secreted factors including exosomes and EVs and not simply through differentiation [87]. Indeed, up to 80% of the therapeutic activities of adult stem cells in infarcted hearts have been shown to be through paracrine mediated effects [88], giving rise to the novel notion of stem cell therapy without the cells [87].

Attractive features of exosomes as noncellular therapeutic candidates include the fact that they are cell permeant and that their membrane protects their molecular cargoes from degradation in the extracellular space. It has been shown that benefits of CDC-derived exosomes injected into ischemic hearts are comparable to those of their parent cells [61]. Recent data support the use of a repeated dose treatment strategy with allogeneic CDC-derived EVs. This provides great flexibility in the further development of this novel cell-free regenerative therapy [86]. The lack of a robust immune response may enable safe and effective repeated dosing of CDC-derived EVs, a desirable feature in the treatment of chronic diseases. Thus, EV-based therapies can potentially circumvent some of the limitations associated with cell transplantation including concerns over immune sensitization with repeated dosing and use of allogeneic cells [86, 89, 90]. Future studies need to further compare the benefits of EV-based therapy with those associated with the delivery of the corresponding EV-producing cells, as well as confirming the safety of allogeneic EV-based therapy. Disease conditions may influence the molecular content of EVs and therefore their therapeutic potency. EVs derived from healthy, young allogeneic donors may represent superior therapeutic products in this regard. Moreover, cells may be modified* in vitro* in order to produce EVs with enhanced beneficial activities. Finally, exosomes may be extremely useful as drug delivery systems [91–93]. For all these reasons, exosomes may help streamline translational applications in many fields of medicine.
